# Administration of Adipose Derived Mesenchymal Stem Cells and Platelet Lysate in Erectile Dysfunction: A Single Center Pilot Study

**DOI:** 10.3390/bioengineering6010021

**Published:** 2019-03-05

**Authors:** Vassilis Protogerou, Efstathios Michalopoulos, Panagiotis Mallis, Ioanna Gontika, Zetta Dimou, Christos Liakouras, Catherine Stavropoulos-Giokas, Nikolaos Kostakopoulos, Michael Chrisofos, Charalampos Deliveliotis

**Affiliations:** 1 Department of Anatomy and Surgical Anatomy, Medical School of Athens, National and Kapodistrian University, 12462 Athens, Greece; vassilis_protogerou@hotmail.com; 2 2nd Urological Department, Attikon Hospital, Medical School of Athens, National and Kapodistrian University, 12462 Athens, Greece; cliakour@theol.uoa.gr (C.L.); medkost@hotmail.com (N.K.); mxchris@yahoo.com (M.C.); 3 Hellenic Cord Blood Bank, Biomedical Research Foundation Academy of Athens, 4 Soranou Ephessiou Street, 11527 Athens, Greece; pmallis@bioacademy.gr (P.M.); giannagont@gmail.com (I.G.); zdimou@bioacademy.gr (Z.D.); cstavrop@bioacademy.gr (C.S.-G.); 4 2nd Urological Department, Medical School of Athens, National and Kapodistrian University, 12462 Athens, Greece; nikistra@hol.gr

**Keywords:** erectile dysfunction, MSCs, stem cells, platelet lysate, IIEF-5 questionnaire

## Abstract

Erectile dysfunction (ED) affects more than 30 million men; endothelial dysfunction plays a significant role in EDs pathogenesis. The aim of this study was to administer mesenchymal stem cells (MSC) derived from adipose tissue and platelet lysate (PL) into patients with erectile dysfunction. This pilot study enrolled eight patients with diagnosed ED. Patients enrolled were suffering from organic ED due to diabetes melitus, hypertension, hypercholesterolaemia, and Peyronie disease. The patients were distributed in 2 groups. Patients in group A received adipose derived mesenchymal stem cells (ADMSC) resuspended in PL while patients in group B received only PL. ADMSCs were isolated from patients’ adipose tissue and expanded. In addition, blood sampling was obtained from the patients in order to isolate platelet lysate. After the application of the above treatments, patients were evaluated with an International Index of Erectile Function (IIEF-5) questionnaire, penile triplex, and reported morning erections. After MSCs and PL administration, patients presented improved erectile function after 1 and 3 months of follow-up. A statistically significant difference was observed in the IIEF-5 score before and after administration of both treatments after the first month *(p* < 0.05) and the third month (*p* < 0.05). No statistically significant difference was observed in the IIEF-5 score between group A and B patients. All patients were characterized by improved penile triplex and increased morning erections. No severe adverse reactions were observed in any patient except a minor pain at the site of injection, which was in the limits of tolerability. The results of this study indicated the satisfactory use of MSCs and PL in ED. MSCs in combination with PL or PL alone seems to be very promising, especially without having the negative effects of the current therapeutic treatment.

## 1. Introduction

Erectile dysfunction (ED) is a common pathology in men and it is estimated that 30 million men are suffering from a different degree of this pathology [1]. It is estimated that 50% of men ages 40 to 70 years will develop ED in the near future [1,2]. ED is the inability to attain or maintain satisfactory penile erection for sexual intercourse. In this way, ED affects a man’s quality of life, as well as his partners’ [3]. 

The corpora cavernosa plays a significant role in establishing an erection [4]. Corpora cavernosa consists of a lattice of sinusoids, which are covered by a single layer of endothelial cells (ECs), multiple layers of circular and longitudinal oriented cavernous smooth muscle cells (CSMCs), and the cavernous nerves (CNs) [4,5,6]. Upon stimulation, the activated neuronal NOS (nNOS) produces NO that leads to the relaxation of the cavernosal smooth muscle cells (CSMCs), which in turn let the blood to fill up the sinusoids [4,7]. The increased intracavernosal pressure compresses the penile veins against the tunica albuginea further decreasing the blood outflow and helps achieve a full erection, which is maintained by the NO produced by the endothelial nitric oxide synthase (eNOS) [4,7]. Furthermore, ECs are expressing significant levels of NO during intercourse, which maintain CSMCs in this induced state [4,7].

Due to damage in key components of erections, such as endothelial cells (ECs), cavernous smooth muscle cells (CSMCs) and neuronal cells, ED may occur [4,8,9,10]. Possibly, during radical prostatectomy, cavernous nerves could be damaged, causing long-term consequences that include diminished production of NO, atrophy of CSMCs, and ECs. This atrophy also could induce penile fibrosis and ECs and CSMCs apoptosis, resulting to the development of penile fibrosis [4]. ED may also occur in men diagnosed with diabetes melitus (DM), a chronic disease that affects more than 371 million people worldwide. DM may also impact and damage both the macrovascular and microvascular systems. Further, it is estimated that men diagnosed with DM have a threefold increased risk for ED [11]. 

Currently, ED treatments include the use of various pharmaceutical agents. The most widely used pharmaceutical agents are phosphodiesterase type-5 inhibitors (PDE5-I) [7]. Although, in some patients a single dose of PDE5-I might obtain or maintain a successful erection upon a sexual stimulus, some patients need to have repetitive doses of these agents in order to acquire or maintain a successful erection. However, if the dosage is not correct in the aforementioned agents, they can be accompanied by adverse reactions. Although some patients are having complicated health issues such as cardiovascular disease (CVD) and DM, their suitability can be reduced significantly [4,7]. In addition, the use of the pharmaceutical agents cannot be considered as curative, since the patients need to use them before sexual intercourse. Under this scope, alternative strategies must be found in order to manage properly or even treat ED. For this purpose, mesenchymal stromal cells (MSCs) could be good candidates for the treatment of ED [3].

MSCs can be isolated by several sources of human body, including bone marrow (BM), adipose tissue (AT), Wharton’s Jelly (WJ) tissue, umbilical cord blood (UCB), and neonatal teeth [12,13,14]. MSCs are known for their immunoregulatory and immunosuppressive functions and have been administrated in patients with autoimmune disorders such as multiple sclerosis (MS), amyotrophic lateral sclerosis (ALS), and Chron’s disease. During the last century, a great effort has been performed to establish their regenerative properties, by using them in tissue engineering and regenerative medicine approaches [12,13,14]. In addition, MSCs have the capability of multipotential differentiation to other lineages such as “osteogenic”, “adipogenic”, and “chondrogenic”. Moreover, several research groups indicate the possible differentiation of MSCs towards “neuronal” cell lineages [12,13,14,15,16]. 

Recently, injections of autologous PL are used in regenerative medicine approaches with promising results. PL contains a significant number of growth factors such as platelet derived growth factor (PDGF), transforming growth factor—β11 (TGF-β11), vascular endothelial growth factor (VEGF), epidermal growth factor (EGF), platelet derived angiogenesis factor (PDAF), and insulin like growth factor (IGF), which is derived from platelets [15,16]. The clinical efficacy of PL depends on the concentration of growth factors that may act as transmitters, inducing wound healing and regeneration of damaged tissues [17,18].

The aim of this study was to define and quantify any improvement in erectile function of ED patients injected with ADMSCs suspended in PL or PL only. Furthermore, the collected data could be used to evaluate the feasibility of the treatment, to define any potential side effects and to estimate the sample size that is needed to design properly the next step, which is the performance of clinical trial.

## 2. Materials and Methods

### 2.1. Study Design

This study is a prospective phase 1, single center pilot study, which has been approved by the Scientific Committee of Attikon Hospital (Ref No 006), Athens, Greece. All patients were informed of the study design and signed a written informed consent in accordance to Helsinki declaration. 

**Figure 1:**
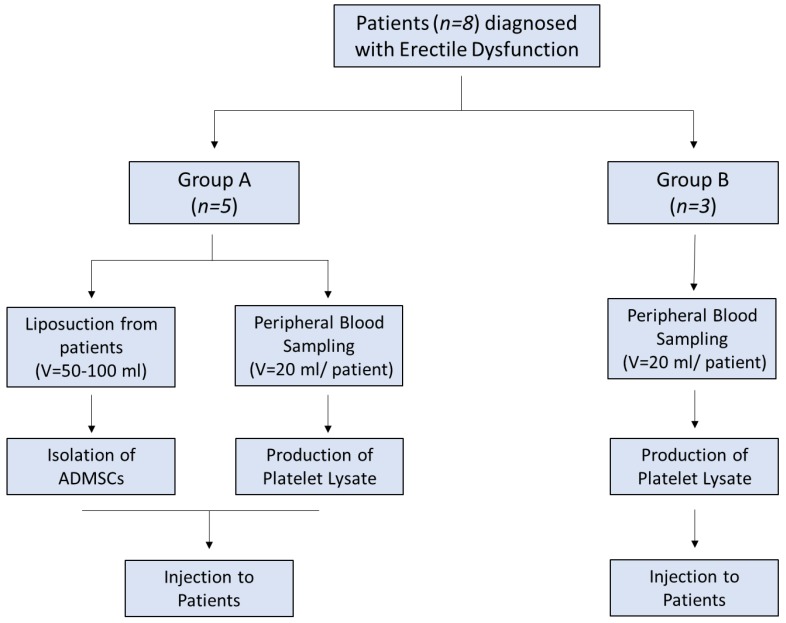
Study overview.

The patients were divided into two groups. Group A involved patients (*n* = 5) who received adipose derived MSCs (adipose derived mesenchymal stem cells (ADMSC)) with PL and group B involved patients (*n* = 3) who received only PL (Figure 1). Inclusion criteria were organic ED due to diabetes mellitus, hypertension, hypercholesterolaemia, and Peyronie disease. Detailed descriptions of each patient characteristics are listed in Table S1. Hormonal and metabolic evaluation were performed in all patients and included testosterone, estradiol, LH, FSH, PRL, FT3, FT4, TSH, α-FP, CEA, CA 19-9, glucose, cholesterol, triglycerides, and PSA (Table S2). In addition, CT scans of the abdomen, thorax, and brain was performed in all patients in order to exclude other pathologies. Evaluation of ED was performed by penile triplex with intracavernosal injection (ICI) of vasodilators and a thorough IIEF-5 questionnaire. 

Specifically, penile triplex was performed using a Doppler ultrasonography device (Chison Qbit7 Ampronix, Medical Imaging Software, Milpitas, CA, USA). Ultrasonograms were obtained in all patients before and after the administration of ADMSCs with PL or PL monotherapy. Each patient was injected with 20 μg of alprostadil (Pfitzer, New York, NY, USA) to cause vasodilation in penile vasculature, thus resulting in an erection. Peak Systolic Velocity (PSV) and End Diastolic Velocity (EDV) were measured every 5 min for a total period of 20 min. PSV and EDV both were measured in cm/s. 

Exclusion criteria for patients were lack of sexual interest, neurologic or hormonal ED, penis injuries others than Peyronie’s disease, and all cases of cancer. Moreover, all patients enrolled in this pilot study were instructed not to stop or change the medication they used for ED during the follow-up period. Each patient’s medication for ED is listed in Table S3. According to the findings, during the evaluation period, their medication was properly adjusted.

### 2.2. Isolation and Expansion of ADMSCs

Lipoaspiration was performed from all patients of group A in order to isolate ADMSCs. Isolation of ADMSCs was performed in compliance with Good Manufacturing Practices (GMPs) at clean rooms provided by the Hellenic Cord Blood Bank (HCBB) of Biomedical Research Foundation Academy of Athens (BRFAA). Briefly, adipose tissue from lipoaspiration was extensively washed in Phosphate Buffer Saline 1× (PBS 1×, Gibco, Life Technologies, Grand Island, NY, USA) for blood removal. Then, the supernatant was removed and enzymatically treated with equal volume of collagenase 1 mg/mL (Sigma-Aldrich, Darmstadt, Germany) at 37 °C in orbital shaker for a maximum of 3 h. Inactivation of collagenase was performed with the addition of PBS 1×, followed by centrifugation at 500 g for 6 min. The supernatant was discarded, the pellet was resuspended in complete cell culture medium, and transferred to 25 cm^2^ cell culture flasks (Costar, Corning Life, Canton, MA, USA) in humidified atmosphere.

After 10 days of incubation, the cell cultures were microscopically checked and upon reaching 70–80% confluency, the ADMSCs were detached with 0.25% trypsin- EDTA solution (Sigma-Aldrich, Darmstadt, Germany), washed with PBS 1×, and replated to 75 cm^2^ flasks (Costar, Corning Life, Canton, MA, USA). The same procedure was repeated until the cells reached passage 4. The medium of cell cultures was changed biweekly. Complete culture medium consisted of α-Minimum Essentials Medium (α-MEM, Gibco, Life Technologies, Grand Island, NY, USA) supplemented with 20% v/v Fetal Bovine Serum (FBS, Gibco, Life Technologies, Grand Island, NY, USA) 1% v/v Penicillin/ Streptomycin (Gibco, Life Technologies, Grand Island, NY, USA), and 1% L-glutamine (Gibco, Life Technologies, Grand Island, NY, USA).

### 2.3. Differentiation Potential of ADMSCs

ADMSCs were differentiated to “osteocytes”, “adipocytes”, and “chondrocytes” in order to establish their multilineage differentiation potential. 

ADMSCs (*n* = 5) were differentiated to “osteocytes” using the StemProΤΜ Osteogenic Differentiation kit (Cat. No. A1007201, Gibco, ThermoFischer, Waltham, MA, USA) according to manufacturer’s instructions. Finally, Alizarin red S (Sigma-Aldrich, Darmstadt, Germany) staining was used in order to validate the successful differentiation of ADMSCs to “osteocytes”.

In addition, ADMSCs (*n* = 5) were differentiated to “adipocytes” with the use of StemProTM Adipogenic Differentiation kit (Ca. No. A1007001, Gibco, ThermoFischer, Waltham, MA, USA) according to manufacturer’s instructions. The successful differentiation of ADMSCs into “adipocytes” was established by Oil-Red O (Sigma-Aldrich, Darmstadt, Germany) staining. Furthermore, “chondrogenic” differentiation of ADMSCs (*n* = 5) was performed using StemProTM Chondrogenesis Differentiation Kit (Cat. No A1007101, Gibco, ThermoFischer, Waltham, MA, USA). Finally, Alcian blue staining was performed in order to establish the successful “chondrogenic” differentiation of ADMSCs. 

### 2.4. Growth Kinetics and Cell Viability of ADMSCs

Growth kinetics, including total cell number, cell doubling time (CDT), population doubling (PD), and cell viability were determined in ADMSCs after each passage until passage 5. For this purpose, 2 × 10^5^ ADMSCs (*n* = 5) were seeded in 75 cm^2^ flasks (Costar, Corning Life, Canton, MA, USA). The total cell number of ADMSCs was counted with the use of Neubauer slide (Celeromics, Valencia, Spain). The estimation of cell viability after each passage was performed, using Trypan blue (Sigma Aldrich, St Louis, MO, USA). The determination of total cell number and cell viability of ADMSCs were performed by two different observers. CDT and PD were estimated according to the following Equations:

(1) $$\mathrm{CDT}=\;\frac{\log_{10}\left(N/N0\right)}{\log_{10}\left(2\right)}\;\times \;(T)$$

and

(2) $$\mathrm{PD}=\;\frac{\log_{10}\left(N/N0\right)}{\log_{10}\left(2\right)}\;$$

where *N* was the number of cells at the end of the culture, *N*0 was the number of seeded cells, and *T* was the culture duration in hours.

### 2.5. Immunophenotypic Analysis of ADMSCs

The immunophenotypic analysis of ADMSCs was performed according to the following panel of monoclonal antibodies. Specifically, ADMSCs (*n* = 3) at passage 4 were analyzed for CD90, CD105, CD73, CD29, CD19, CD31, CD45, CD34, CD14, CD3, CD19, HLA-DR and HLA-ABC. The CD90, HLA-ABC, CD29, CD19, CD31, and CD45 were fluorescein isothiocyanate (FITC) conjugated, while CD105, CD73, CD44, CD3, CD34, CD14, and HLA-DR were phycoerythrin conjugated. All monoclonal antibodies were purchased from Immunotech (Immunotech, Beckman Coulter, Marseille, France). The immunophenotypic analysis was performed in Cytomics FC 500 flow cytometer coupled with CXP Analysis software (Beckman Coulter, Marseille, France).

### 2.6. Preparation of Platelet Lysate

A total of 20 mL of peripheral blood was received from patients of group A (*n* = 5) and group B (*n* = 3) in order to isolate platelet rich plasma (PRP). The blood sampling was performed in 5 mL citrate phosphate dextrose adenine (CPDA) treated vacutainer tubes. Then, centrifugation was performed at 160 g for 20 min, followed by the isolation of plasma layer which contained the platelets (PLTs). A second centrifugation was performed at 420 g for 15 min. Finally, the volume of PRP was reduced in order to obtain the desired platelet number. PRP was stored at −80 °C for 48 h. Upon use, PRP was thawed, forming the PL, which was finally injected to the patients. 

### 2.7. ADMSCs and PL Administration

ADMSCs were trypsinized, centrifuged, and the cell pellet was resuspended in 2 mL of PL in group A. Patients of group B were injected only with PL. Prior to injection, the base of penis was clamped and remained for a time period of 10 min. ADMSCs resuspended in PL or PL only were injected directly in corpora cavernosum of penis. After the injection, the patients were reevaluated on the first and third of the month. Patients follow up included physical and andrological evaluation, IIEF-5 questionnaire, and penis triplex. 

### 2.8. Statistical Analysis 

In this initial pilot study, no sample size calculations were conducted. Statistical analysis was performed with Graph Pad Prism v 6.01 (GraphPaD Software, San Diego, CA, USA). IIEF-5 scores were analyzed by Friedman’s test for multiple non-parametric comparisons and then Mann-Whitney test was also applied. Statistically significant difference between group values was considered when *p* value was less than 0.05. Indicated values were presented as mean ± standard deviation.

## 3. Results 

### 3.1. Characterization of ADMSCs

**Figure 2:**
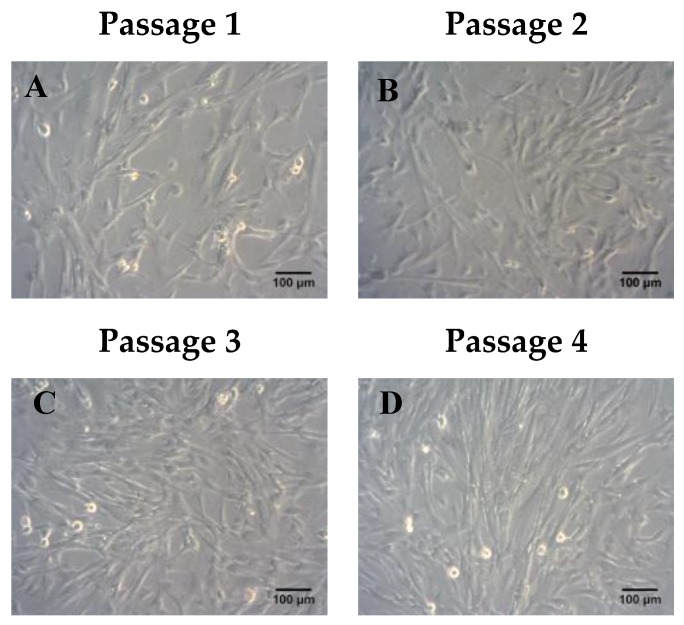
Morphological characteristics of adipose derived mesenchymal stem cells (ADMSC) until they reached passage 4. Representable images of ADMSCs from passage 1–4 (**A**–**D**). Scale bars 100 μm.

**Figure 3:**
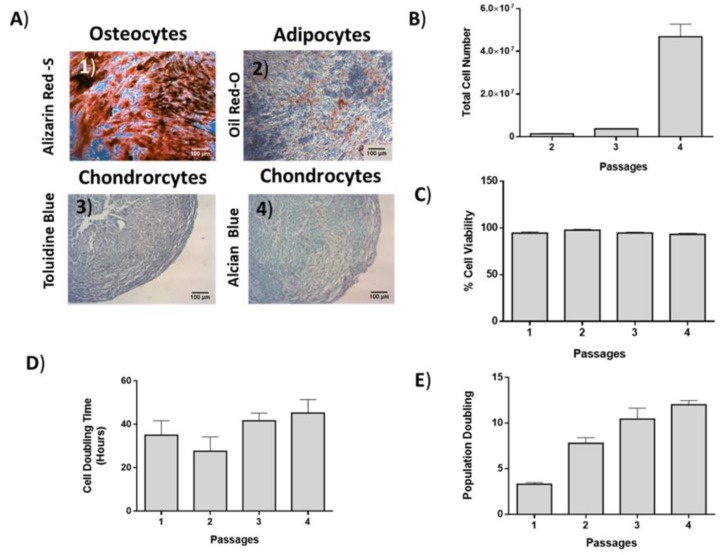
Differentiation potential, growth kinetics and cell viability of ADMSCs. (**A**) ADMSCs were successfully differentiated to “osteocytes”, “adipocytes”, and “chondrocytes”. Scale bars 100 μm. (**B**) Total cell number, (**C**) cell viability, (**D**) cell doubling time (CDT), and (**E**) population doubling (PD) of ADMSCs.

ADMSCs were characterized by fibroblastic morphology, which was retained until passage 4 (Figure 2). In addition, ADMSCs were successfully differentiated into “osteocytes”, “adipocytes”, and “chondrocytes”. Specifically, ADMSCS differentiated from “osteocytes” and exhibited calcium deposits, which were visible by the Alizarin Red S staining (Figure 3A). Furthermore, the produced lipid droplets of the differentiated “adipocytes” were stained successfully by the Oil Red O staining (Figure 3A). Finally, the ADMSCs were capable to be differentiated into “chondrocytes” as indicated by the histological stains Toluidine and Alcian blue (Figure 3A). 

The average cell number of ADMSCs at passage 4 was 47 × 10^6^ (Figure 3B). The average CDT and PD of ADMSCs at passage 4 was 45 ± 6 h and 12 ± 0.46, respectively (Figure 3D,E). 

### 3.2. Immunophenotypic Analysis

**Figure 4:**
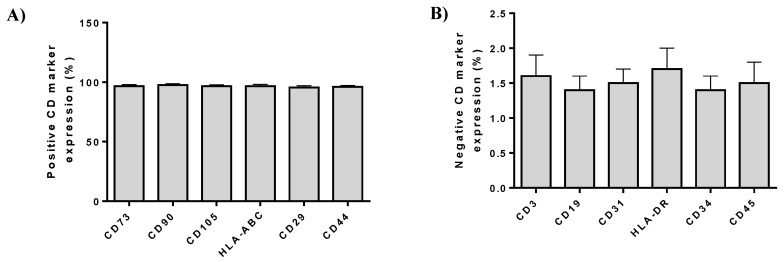
(**A**) Immunophenotypic analysis of ADMSCs by flow cytometer. Positive expression (%) of cell doubling (CD) markers in ADMSCs. (**B**) Negative expression (%) of CD markers in ADMSCs.

Flow cytometric analysis showed the positive expression of ADMSCs were positive (over 95%) for CD73, CD90, CD105, HLA-ABC, CD29, and CD44 and negative (below 2%) for CD3, CD19, CD31, HLA-DR, CD34, and CD45 (Figure 4).

### 3.3. Production of PL

**Figure 5:**
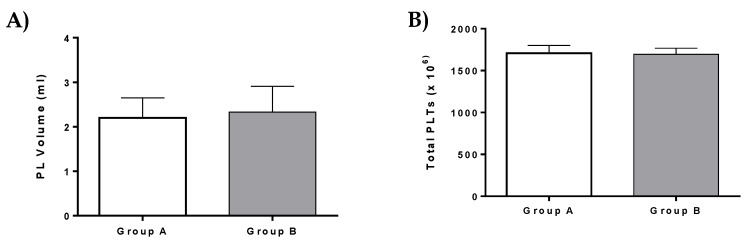
PL volume and total platelets (PLTs) of patients. (**A**) Platelet lysate (PL) volume of patients from group A and B. (**B**) Total number of PLTs of patients from group A and B. No statistically significant difference was observed in PL volume and total number of PLTs between group A and group B (*p* > 0.9).

PL was successfully isolated from all patients of the current study. Specifically, a total volume of 2.2 ± 0.3 mL of PL which contained 1708 ± 76 × 10^6^ PLTs was isolated and used in patients of group A (Figure 5). In group B, a total volume of 2.3 ± 0.4 mL PL with 1693 ± 52 × 10^6^ PLTs was isolated (Figure 5). A detailed description of PL characteristics is listed in Table S4. No statistically significant difference was observed either in PL volume or total PLTs between group A and B. 

### 3.4. Patient’s Follow-up

Patients of group A received 38.9 ± 14.4 × 10^6^ ADMSCs in combination with 2.2 ± 0.3 mL of PL. A detailed description of ADMSCs administration to each patient is presented in table S4. Prior to ADMSCs administration, all patients were unable to have successful intercourse without the use of oral PDE5i or ICI. Following ADMSCs administration, the erectile function was improved in all patients. The majority of patients in group A were characterized by an increase in IIEF-5 score after three months of evaluation (Table 1). In addition, the patients were characterized by an improved trend on Peak Systolic Velocities (PSV), while there was a more variable pattern on the End Diastolic Velocities (EDV) as it is indicated in Table 2. Patient 1 and 2 after ADMSCs administration reported morning erections, successful sexual intercourse and their medication changed from ICI (prior to treatment) to PDE5i. Patient 3 did not present any improvement in IIEF-5 score, although he had improvement in morning erections, his medication changed from ICI (prior treatment) to PDE5i. In addition, patient 4 after the first month of ADMSCs administration noticed morning erections, while patient 5 noticed morning erections after three months. These patients achieved to maintain satisfactory unassisted erections during the whole time of sexual intercourse. 

Patients of group B (Patients 6–8) received 2.3 ± 0.4 mL of PL. All patients in this group experienced an increase in PSV, EDV, and IIEF-5 score after the first and third month of evaluation. All patients reported improved erectile function and noticed morning erections. Moreover, patients were able to have unassisted erections that were maintained during sexual intercourse. Patient 6 tried several treatments before PL administration including PDE5i, ICI, and shock wave, but his erections did not improve. Following PL administration, this patient was able to have unassisted erection for intercourse in 70% of the times. Patient 7 achieved a satisfactory erection, which was maintained successfully during sexual intercourse. Patient 8 reported improved erectile function and achieved to have unassisted intercourse in 30% of the times. All patients reported improved morning erections. In addition, the pharmaceutical treatment of these patients was gradually reduced. Their erectile function was not altered, thus having successful sexual intercourse during the three months of evaluation.

**Figure 6:**
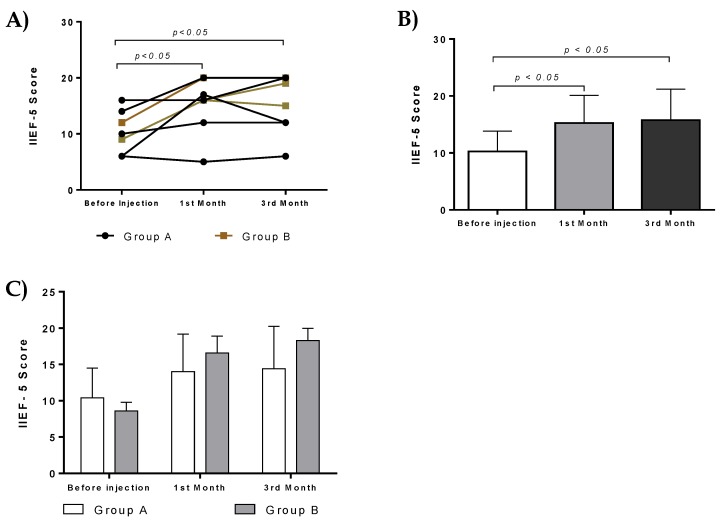
Effect of ADMSCs and PL therapy. IIEF scores of each patient from group A and B after intracavernous injections. (**A**) Patients improved their erectile function and had statistically significant IIEF scores after the first and third months. (**B**) IIEF score of all patients received intracavernous injections. Statistically significant difference in IIEF score of patients before the initiation of treatment and first (*p* < 0.05) and third (*p* < 0.05) month. (**C**) IIEF score from patients of group A and B.

There was statistically significant difference in IIEF-5 score in both groups before and after administration. Specifically, statistically significant difference was observed in IIEF-5 score before and after administration (first month, *p* < 0.05, and 3rd month, *p* < 0.05, Figure 6A,B). No statistically significant difference was observed in IIEF-5 score between patients of group A and B (Figure 6C).

### 3.5. Side Effects

No significant side effects or complications during administration of ADMSCs with PL or PL alone were reported by the patients from both groups. All patients felt a minor pain at the time of injection. The pain was more intense in group B patients and resolved spontaneously after a short time. During follow-up, no adverse reactions were reported. 

## 4. Discussion

ED globally affects a large number of adult men and significantly reduces their quality of life. Current treatments involved the use of PDE5-I, which induces mild side effects in 70% of patients. Moreover, the use of PDE5-I is limited in patients with cardiovascular disease and diabetes melitus [4,7]. Second line treatments for ED involved the use of vacuum devices and intrecavernosal injections with PDE5-I [11]. To date, all these approaches have only temporal effects in patients suffering from ED. Recently, stem cell therapy and its derivatives have gained great attention in reversing pathological states and therefore may act as a potent ED treatment [3].

The aim of this study was to assess the safety and efficacy of the current prospective treatment in ED. In this study, autologous ADMSCs supplemented in PL and PL monotherapy were used for the treatment of ED. 

Specifically, intracavernous injections of ADMSCs with PL or PL alone in patients from both groups were feasible, safe, and well-tolerated. No serious adverse events were reported after three months of patients follow-up. On physical examination, no alteration in body temperature, blood pressure, heart rate, and respiratory rate was observed. 

In this study, two different groups were used. In group A, injection of ADMSCs resuspended in PL was applied, while group B involved patients that were injected only with PL. ADMSCs were characterized by fibroblastic morphology, successfully differentiated to “osteocytes”, “adipocytes”, and “chondrocytes”, expressed CD73, CD90, and CD105 over 95%, while lacking the expression of CD34, CD45, and HLA-DR, fulfilled in this way the minimal criteria of International Society for Cellular Therapy [14].The data indicated that ADMSCs were not characterized by different properties compared to other MSCs sources, such as Wharton’s Jelly tissue, umbilical cord blood, and bone marrow. Moreover, no extended invasive surgical procedures are required for the isolation of the adipose tissue. Typically, from 1 g of adipose tissue, more than 3.5 × 10^5^ MSCs can be isolated and used immediately or after expansion, in regenerative medicine approaches [12,13].

ADMSCs reached passage 4 successfully and were negative for aerobic, anaerobic, and mycoplasma contamination. The above data indicated that ADMSCs was a properly defined cell population, processed under GMPs conditions. In the literature, MSCs have been used successfully for treatment of various human disorders including autoimmune diseases [12,13]. In addition, properly defined MSCs secrete a variety of growth factors and cytokines which are contributing to tissue regeneration [12]. Moreover, it has been shown that despite the age discrepancy between patients, ADMSCs were successfully isolated and expanded without significant alteration in their CDT, PD, and cell viability.

This study also involved the administration of PL in patients with ED. Specifically, PL that was used in patients of group A and group B did not present any significant alteration either in the number of isolated PLTs or the injected volume. In addition, PL possesses a rich source of growth factors, which can be derived efficiently from patient’s peripheral blood [16,17,18]. To date, in the literature, PL has been used in various regenerative medicine approaches. 

After administration and three months of follow-up assessment, the majority of patients from both groups were characterized by improved IIEF-5 score, penile triplex, and a greater number of morning erections. All patients from both groups were able to have successful sexual intercourse. The medication of three patients of group A was changed from ICI to oral PDE5i. In addition, two patients from group A and all patients from group B had unassisted satisfactory erections. In these patients the pharmaceutical treatment was reduced gradually during the three months of evaluation. Despite their reduced treatment, these patients were able to perform successful sexual intercourse. Indeed, statistically significant differences in IIEF-5 score were found either in group A or B, before and after treatment (first and third month). In addition, group B presented higher IIEF-5 score in comparison to group A, although no statistically significant difference was found between these two groups. Patients in both groups experienced improvement after treatment. Although there is no statistical difference between groups, patients in group B showed improvement in their IIEF score and penile triplex results. This fact implies that PL monotherapy seemed to have better results in ED compared to ADMSCs administration. More investigation is needed to be performed in order to safely conclude which approach might have a better outcome in patients with ED.

The improvement of erectile function in patients from both groups may be as a result to the paracrine effects both of ADMSCs and PL. There is an increasing number of studies, where the MSCs have been administrated, improving the condition in various pathologies such as osteoarthritis, bone and cartilage damage, and even autoimmune disorders including Crohn’s disease, multiple sclerosis, and ALS [19,20,21,22]. On the other hand, platelet lysate and its containing growth factors have been reported previously for their successful use in wound and burn healing [15,16]. In this way, both treatments may rescue endothelial dysfunction, which is common in ED, elevating the production of NO, thus improving the overall erectile function [4,8,9,10]. In our study, due to an underlying disease of the patients such as diabetes, hypertension, and hypercholesterolaemia, the endothelial dysfunction might be a possible explanation, and this might explain the improvement of post treatment. However, more research must be performed towards this way, in order to obtain safe results regarding the underlying therapeutic mechanism.

The results of our study seemed to be in accordance with previous published reports [23,24,25,26,27,28]. In the current study, the safety and efficacy of ADMSCs and PL injection were assessed. On the other hand, in the study of Bahk et al. [26], where single intracavernous injections of allogeneic umbilical cord blood stem cells were performed, no safety regarding the intracavernous injections was assessed. Moreover, in the current study, the patients did not perform any radical prostatectomy, but were characterized by other disorders such as hypertension and high blood glucose levels. However, the administration of ADMSCs resuspended in PL or the administration with PL only seemed to have a positive effect in erectile function, as has been reported by others [23,24,27,28].

This study was also characterized by several limitations in its performance. This pilot study was unblinded lacking the control group. In addition, group B involved only three patients. In order to obtain safe conclusions regarding the regenerative potential of ADMSCs and PL, further evaluation must be performed including follow up assessment after 6 and 12 months.

## 5. Conclusions

The results of the current study were very promising regarding the function improvement of ED patients. ADMSCs resuspended in PL or only PL injections could positively contribute to the treatment of ED. After three months of follow-up, patients injected only with PL seemed to have comparable outcome to patients that were treated with ADMSCs resuspended in PL. Further evaluation must be performed in order to safely conclude which approach might have the best outcome in patients with ED.

The future goal of this study is to enroll a higher number of patients who will be evaluated for their erectile function over a longer time period of time. ED compromise a wide socioeconomic burden, affecting a great number of men, and any possible therapeutic strategy without the adverse effects of previous treatments may be very promising.

## Figures and Tables

**Table 1:** IIEF-5 scores.

	Patient	Before Administration	1st Month	3rd Month
Group A	1	6	17	12
	2	10	12	12
	3	6	5	6
	4	14	20	22
	5	16	16	20
Group B	6	12	20	20
	7	9	16	19
	8	9	16	15

**Table 2:** Penile triplex results.

	Patient	Before Administration (cm/s)	1st Month (cm/s)	3rd Month (cm/s)
Group A	1	35/11	30.5/7.8	39/12
	2	40.3/8.6	25.4/6.0	40.8/11.0
	3	16.1/4.7	35/8.9	61.2/20.6
	4	57/15	78.2/16.6	97.9/22
	5	45.5/17.8	49.4/14.7	62.6/25.8
Group B	6	69.9/18.7	81.9/13.4	79/12.7
	7	40/0	52.7/−10	101.5/0
	8	60/0	65.9/6.9	63.1/−7.6

## Supplementary Materials

The following are available online at https://www.mdpi.com/2306-5354/6/1/21/s1, Table S1. Patient’s age and comorbidities. Table S2. Hormonal and metabolic evaluation of all patients. Table S3. Patients’ medication before the administration of ADMSCs with PL or PL. Table S4. Number of administrated ADMSCs to each patient.
