# Supplementary material for: Administration of Adipose Derived Mesenchymal Stem Cells and Platelet Lysate in Erectile Dysfunction: A Single Center Pilot Study
Source: Bioengineering (Basel). 2019 Mar 5;6(1):21. doi: 10.3390/bioengineering6010021 (PMC6466012; doi:10.3390/bioengineering6010021)
Supplement: Supplementary file 1 [file bioengineering-06-00021-s001.pdf]

Table S1. Patient's age and comorbidities.

	Patient No	Age	Comorbidities
Group A	1	62	Hypertension, Hypercholesterolemia
	2	52	Diabetes, Hypercholesterolemia
	3	52	Diabetes, Hypertension
	4	66	Peyronie's Disease
	5	52	Diabetes, Hypertension, Hypercholisterolaimia
Group B	6	39	Peyronie's Disease
	7	39	Peyronie's Disease
	8	33	Hypertension

Table S2. Hormonal and metabolic evaluation of all patients.

	Group A					Group B		
**Parameters**	**Patient 1**	**Patient 2**	**Patient 3**	**Patient 4**	**Patient 5**	**Patient 6**	**Patient 7**	**Patient 8**
**LH** (mIU/ml)	4.8	4.8	5.8	6.3	3	3.4	4.5	2.3
**FSH** (mIU/ml)	4.8	6.1	10.6	5.3	6.1	5.1	6.3	6.6
**PRL** (ng/ml)	7.2	8	8.2	13.1	5.6	8.3	9.1	14
**FT3** (pg/ml)	2.4	2.5	2.9	2.7	2.8	2.9	2.8	2.2
**FT4** (ng/dl)	1.1	0.9	1.2	1.4	1	0.9	1.2	1.2
**TSH** (μIU/ml)	2.2	1.9	2.2	0.6	1.9	2.5	3.1	0.7
**CEA** (IU/ml)	1.1	0.8	0.9	1.2	1.6	1.7	1.8	1.7
**CA 19-9** (IU/ml)	9.1	9.1	7.8	10.1	11.1	9.5	7.2	7.8
**aFP** (ng/ml_	1.8	2.1	1.1	1.7	2.1	1	1.6	1.7
**PSA** (ng/ml)	0.4	0.3	0.9	0.9	0.4	0.9	0.8	1.3
**Glucose** (mg/dl)	98	91	269	114	191	191	88	95
**Cholesterol** (mg/dl)	180	140	171	210	151	169	172	168
**Triglycerides** (mg/dl)	323	175	242	126	59	89	122	85
**Testosterone** (nmol/L)	5.9	6.6	2.9	4.9	5.5	3.1	3.4	9.5
**Estradiol** (mg/dl)	53.1	35.2	31.5	45.1	37.5	25.4	22.5	11.5

Table S3. Patients' medication before the administration of ADMSCs with PL or PL

	Patient	Erectile Function before therapy	Erectile function 1st month	Erectile function 3rd month
Group A	1	Only with ICI, unable to climax	Hard erections with oral PDE5-I, normal ejaculation	Hard erections with oral PDE5-I, normal ejaculation
	2	No erections	Some increase in hardness	Hard erections with oral PDE5-I
	3	Moderate erections with ICI	Improvement only in morning erections	Hard erections with oral PDE5-I
	4	Moderate erections with ICI	Hard erections with oral PDE5-I	Unassisted hard erections
	5	Hard erections only with ICI	Unassisted hard erections short duration	Unassisted hard erections
Group B	6	Hard erections with PDE5i	Unassisted hard erections on 70% of intercourses	Unassisted hard erections in 70% of intercourses
	7	Moderate erections with PDE5i	Improvement-still on PDE5i	Stable
	8	Hard erections only with ICI	Unassisted hard erections of short duration	Good Unassisted hard erections in 30% of intercourses

Table S4. Number of administrated ADMSCs to each patient

	Patient No	Tissue Specimen	Number of injected ADMSCs (x 10^6 cells)	Volume of PRP (ml)	Total PLTs (x 10^6)
**Group A**	1	Lipoaspiration, 20 ml	9.5	2	1600
	2	Lipoaspiration, 50 ml	43.2	2	1700
	3	Lipoaspiration, 100 ml	37.2	2	1728
	4	Lipoaspiration, 50 ml	53.2	2	1660
	5	Lipoaspiration, 100 ml	51.4	3	1850
**Group B**	6	-	-	2	1750
	7	-	-	3	1610
	8	-	-	2	1720
